# MiR200 and miR302: Two Big Families Influencing Stem Cell Behavior †

**DOI:** 10.3390/molecules23020282

**Published:** 2018-01-30

**Authors:** Francesca Balzano, Sara Cruciani, Valentina Basoli, Sara Santaniello, Federica Facchin, Carlo Ventura, Margherita Maioli

**Affiliations:** 1 Department of Biomedical Sciences, University of Sassari, Viale San Pietro 43/B, 07100 Sassari, Italy; mariafrancesca22@virgilio.it (F.B.); sara.cruciani@outlook.com (S.C.); valebasoli@gmail.com (V.B.); sara.santaniello@gmail.com (S.S.); 2 Laboratory of Molecular Biology and Stem Cell Engineering, National Institute of Biostructures and Biosystems, Innovation Accelerator, CNR, Via Piero Gobetti 101, 40129 Bologna, Italy; ventura.vid@gmail.com; 3 Department of Experimental, Diagnostic and Specialty Medicine (DIMES), University of Bologna, Via Massarenti 9, 40138 Bologna, Italy; federica.facchin2@unibo.it; 4 GUNA ATTRE (Advanced Therapies and Tissue REgeneration), Innovation Accelerator, CNR, Via Piero Gobetti 101, 40129 Bologna, Italy; 5 Istituto di RicercaGenetica e Biomedica, Consiglio Nazionaledelle Ricerche (CNR), Monserrato, 09042 Cagliari, Italy; 6 Center for Developmental Biology and Reprogramming (CEDEBIOR), Department of Biomedical Sciences, University of Sassari, Viale San Pietro 43/B, 07100 Sassari, Italy

**Keywords:** stem cells, miRNA, epigenetics, cell reprogramming

## Abstract

In this review, we described different factors that modulate pluripotency in stem cells, in particular we aimed at following the steps of two large families of miRNAs: the miR-200 family and the miR-302 family. We analyzed some factors tuning stem cells behavior as TGF-β, which plays a pivotal role in pluripotency inhibition together with specific miRNAs, reactive oxygen species (ROS), but also hypoxia, and physical stimuli, such as ad hoc conveyed electromagnetic fields. TGF-β plays a crucial role in the suppression of pluripotency thus influencing the achievement of a specific phenotype. ROS concentration can modulate TGF-β activation that in turns down regulates miR-200 and miR-302. These two miRNAs are usually requested to maintain pluripotency, while they are down-regulated during the acquirement of a specific cellular phenotype. Moreover, also physical stimuli, such as extremely-low frequency electromagnetic fields or high-frequency electromagnetic fields conveyed with a radioelectric asymmetric conveyer (REAC), and hypoxia can deeply influence stem cell behavior by inducing the appearance of specific phenotypes, as well as a direct reprogramming of somatic cells. Unraveling the molecular mechanisms underlying the complex interplay between externally applied stimuli and epigenetic events could disclose novel target molecules to commit stem cell fate.

## 1. Introduction

Epigenetics is a physiological phenomenon of heritable changes in gene function that do not involve changes in the DNA sequence [1]. These changes of cellular and physiological phenotypic traits may result from external or environmental factors, or they may be part of a normal developmental program. In the last years, researchers have focused on the intertwining of epigenetic changes and stem cell dynamics. One of the most extraordinary mechanism that has been described to influence epigenetic processes is the involvement of non-coding RNA transcripts [1,2,3,4,5], in particular microRNAs (miRNAs). Epigenetic regulation by miRNAs can influence some functional aspects and the differentiation of stem cells towards different cell lineages. Several studies confirm the important role of miRNAs involved in cell life during differentiation, growth, expansion, and apoptotic processes. Moreover, miRNAs have arisen as critical molecular regulators for maintaining the functions of stem cells by finely tuning the levels of different signaling proteins [4,5]. In stem cells, this requires a massive and rapid transformation in the cellular phenotype, and prompt important changes in the proteomic network. miRNAs are able to suppress the translation of many target mRNAs, thus inducing fluctuations in gene expression [6]. Approximately, thousands of miRNAs have been identified so far, making miRNAs one of the most abundant classes of gene regulatory molecules in animals [7]. Nevertheless, understanding the mechanisms accounting for their function is still a remarkable challenge. In fact, owing to the important role and functions of miRNAs in regulating many cellular decisions related to pathological processes, they could be evaluated as new therapeutic targets for drug development in the treatment of many diseases. Understanding miRNA biogenesis, regulation, and mechanism in controlling stem cell differentiation will enhance the likelihood for success in stem cell-based therapeutic approaches, including tissue regeneration and engineering. Within this context, an interesting pathway is the relationship between miRNAs and TGF-β signaling, which has been extensively investigated. Studies by different authors suggest that TGF-β-related signals can either inhibit or enhance miRNA maturation [8,9], being themselves regulated by these small molecules. The aim of the present review is to present the role of specific miRNAs in regulating stem cell patterning, by influencing their pluripotency and differentiation capability. A section will be dedicated to the influence of specific physical stimuli, as electromagnetic fields, on stem cell epigenetic fate.

## 2. Stem Cells

Stem cells are undifferentiated cells that can differentiate into specialized cells or divide to produce more stem cells. In mammals, there are two broad types of stem cells: embryonic stem cells, which are isolated from the inner cell mass of blastocysts, and adult stem cells, which are found in various tissues. In adult organisms, stem cells and progenitor cells act as a repair system for the body, replenishing impaired tissues, having the remarkable potential to develop into many different cell types.

The key characteristics of stem cells are:

1. Asymmetric divisions with the formation of stem cells (self-regeneration or self-renewal) and daughter cells with reduced differentiation potential, which transiently replicate and thus differentiate after a specific number of divisions;
2. The persistence of the replicative capacity for the entire life of the individual;
3. The maintenance a source of stem cells due to a specific microenvironment (stem cell niche) formed by other cells.

Self-regeneration is the ability to create an undifferentiated cell perfectly identical to the original stem cell (SC). Their cellular power (plasticity or stemness) is indeed the ability to develop into highly specialized cell types for specific biological functions. Depending upon the pluripotency, we can distinguish different types of stem cells. Totipotency is the ability to give rise to all embryonic and extraembryonic tissues, and is essentially restricted to the earliest stages of embryonic life, when the embryo is formed by only four or eight cells. Pluripotency is the ability to differentiate in all embryonic tissues, typical of the embryonic SC (ESCs) and of the induced pluripotent SC (IPSCs). Multipotency the ability of one of the three germinative layers (endoderm, mesoderm, or ectoderm), or a specific tissue, to differentiate into different cell types and is associated with adult SC. Finally, unipotency is the ability to differentiate into a single cell type [10,11].

## 3. Embryonic and Adult Stem Cells. Cellular Reprogramming and Generation of Pluripotent Stem Cells

Human Embryonic stem Cells (hESCs) were described for the first time in 1998 after being isolated from the internal cellular masses of human blastocysts which were donated for research purposes [12]. These cells were characterized, in vivo, through the formation of teratomas and, in vitro, through the expression analysis of specific enzymatic markers and assays, and through differentiation studies [12]. Unlike animal models, it is not possible, for ethical reasons, to perform chimerism studies and transmission to the germ line on hESCs. It is known that stem cells (both human and murine) have the ability to replicate indefinitely in vitro, although there are differences both at the gene expression level and at the growth medium conditions (e.g., the use of the Leukemia Inhibitory Factor—LIF—is indispensable for murine stem cells, but it is not necessary for human ones) [13].

## 4. Induced Pluripotent Stem Cells (iPSCs)

In 2006, Yamanaka, Nobel Prize for Medicine or Physiology in 2012, and his Collaborators at the University of Kyoto identified conditions that could “genetically reprogram” specialized adult cells to assume a state similar to that of stem cells. These adult cells, called induced pluripotent stem cells (iPSCs), have been reprogrammed by introducing important genes able to induce self-renewal and essential differentiation capability, like in ESCs. The strategy used was the nuclear reprogramming, a technique that experimentally induced a stable change in the nucleus of a mature cell, which can be maintained during the following cellular divisions. These changes are most frequently associated with the reacquisition of a pluripotent state, thereby providing the cells with a developmental potential [14]. This approach uses mature “somatic” cells from the adult and introduces the genes encoding critical transcription factors, which in turn regulate other key genes for the early steps in embryonic development. Four transcription factors (Oct4, Sox2, Klf4, and c-Myc) were required to reprogram mouse fibroblasts (cells found in the skin and other connective tissues) to acquire an embryonic-like state [8]. These factors were known to be involved in the maintenance of pluripotency. The newly-created iPSCs were found to be highly similar to ESCs and could be produced after several weeks in culture [8,9]. In 2007, two different research groups reached a new milestone by deriving iPSCs from human cells, using either the original four genes [10] or a different combination containing Oct4, Sox2, NANOG and Lin28 [11,15]. Since then, Researchers have generated iPSCs from somatic tissues of monkeys [14] and rats [16,17]. However, these cells can retain methylation patterns from their original cells, a phenomenon called epigenetic memory, which has been shown to influence the differentiation potential [18]. Several studies analyzed the operating system in iPSCs in the context of reprogramming yield. Armstrong et al. [19] compared iPSCs with ESCs, showing that reactive oxygen species (ROS) levels and mitochondrial mass are similar in these cells and significantly less than in human dermal fibroblasts from which iPSCs were first generated. These cells are expected to reproduce the behavior of ESCs in response to oxidative stress (OS). Armstrong et al. [20] also found a reduced number of mitochondria in pluripotent stem cells, as compared to fibroblasts. The loss of mitochondria appears to be a key step in the reprogramming process, being linked to a switch from an oxidative to a glycolytic-dependent metabolism [21]. It has been shown that to support their high proliferation rates, pluripotent stem cells are predominantly anabolic and tend to inactivate catabolic pathways. Consequently, energy production is obtained through glycolysis rather than oxidative phosphorylation, which is related to the decrease in the number of mitochondria [22,23]. A recent study suggests that some miRNAs play a fundamental role in regulating differentiation and maintaining pluripotency/multipotency in stem cells [24].

## 5. miRNAs

miRNAs are a type of non-coding RNA, 21–24 nucleotides in length, that function in the post-transcriptional regulation of gene expression. They can be found as isolated transcript units or clustered and co-transcribed as polycistronic primary transcripts [25]. Typically a miRNA interacts, through its specific sequence named Seed, with specific mRNAs through complementary base-pairing, to influence the translation or stability of the target mRNA molecule. MiRNA life is subjected to multipurpose regulation [26], beginning with the transcription of its own genes. The resulting transcribed RNA sequence is folded into a hairpin structure and then processed in the nucleus by Drosha (a class 2 ribonuclease enzyme III) in a new structure called pre-miRNA. Exportin-5 exports the hairpin pre-RNA from the nucleus to the cytoplasm. Subsequently, another endonuclease, called Dicer, catalyzes pre-miRNA deployment into mature miRNA. Argonaut and helix proteins are co-recruited to form the RNA-induced silencing complex (RISC) that allows the control of miRNA with consequent translational repression, and destabilization cleavage for post-transcriptional protein regulation. Most targeted miRNA sites are found in the 3′untranslated regions (3′UTR) of mRNA transcripts [27]. However, other studies have shown that targeted miRNA sites may reside beyond 3’UTR in mRNA protein encoding sequences [28]. Some pre-miRNAs can be secreted from the cells in the extracellular environment, becoming circulating miRNAs [29], to exert their role in other cells within the tissue or in different tissues. MiRNAs regulate genes and define cell fate and behavior. One of the main miRNA control and regulation mechanisms is represented by transforming growth factor beta (TGF-β) signaling [8,9].

## 6. Stem Cells, TGF-β, and Epigenetics

TGF-β is a multifunctional cytokine belonging to the transforming growth factor superfamily that includes four different isoforms (TGFB1, TGFB2, TGFB3, and TGFB4) and many other signaling proteins produced by all white blood cell lineages [8,9]. Activated TGF-β recruits other factors to form a serine/threonine kinase complex that binds to the TGF-β receptors, which comprises both type 1 and type 2 receptor subunits. After binding TGF-β, the two receptor subunits undergo a cross phosphorylation, then the activated receptor recruits the signaling cascade of small mother against decapentaplegic (SMAD) proteins [30,31]. This leads to the activation of different downstream targets and regulatory proteins, inducing the transcription of different target genes involved in differentiation, chemotaxis, proliferation, and activation of many immune cells [32]. A defect in the growth inhibitory response to TGF-β often correlates with the malignancy of many types of cancers [33]. Among the TGF-β protein signaling, Activin/Nodal branch is essential for maintaining pluripotency in hESCs [34,35,36]. Components of the hESC core transcriptional regulatory circuitry [37], such as NANOG represent one of the target genes that are activated by Smad2/3 [38,39]. One of the key events in acquiring stem cell properties of both breast cancer and normal mammary stem cells is an epithelial-mesenchymal transition (EMT) induced by TGF-β. TGF-β family signaling regulates stemness of normal and neoplastic stem cells by exerting different effects depending upon the cell type, the microenvironment and physiological state of the cells [40,41]. Recent evidence indicates an association between miRNAs and TGF-β signaling, providing a new insight into the deepness of these interactions that are also the basis of pluripotency and cell differentiation [42,43]. The relationship between TGF-β and miRNA signaling has been extensively studied, unraveling both an inhibitory or stimulatory role of the TGF-β pathway on the maturation of miRNAs [44]. Moreover, Smad proteins have been shown to control the transcription of miRNA coding genes by binding their gene promoter [45]. Smads control miRNAs synthesis by a Smad2-3 complex binding to Smad4 [44]. The Smad2-3-4 complex is moved to the nucleus where it binds to specific Smad binding elements in the DNA, while the Smad3 is recruited from the Drosha/DGCR8 microprocessor complex and promotes miRNA maturation [44,46,47]. The mechanisms that lie at the basis of the suggested activation pathways have not been completely elucidated. Some of the known activation pathways are cell or tissue-specific; others have been seen in response to many cytotoxic agents [48,49]. Some of the factors that can trigger the TGF-β are: proteases, integrins, pH, and ROS (reactive oxygen species) [50,51]. The perturbation of one of these activating factors can lead to altered TGF-β levels which in turn bring several outcomes such as: inflammation, autoimmune reactions, fibrosis, and cancer [52].

## 7. MicroRNAs and Cell Reprogramming

As reported above, Yamanaka and his Co-workers showed the feasibility to obtain pluripotent progenitors from fibroblasts by forcing the expression of four genes: OCT3/4, Sox2, Klf4, and C-MYC, the so called Yamanaka factors or OSKM [53]. This discovery is considered one of the most important of the third millennium since it proved the chance of reversing life trajectories [54,55,56]. Interestingly, some of those stem cell core regulators are able to activate the promoters of several miRNAs in ESCs, including miR-290–295, miR-302-367, and miR-92clusters [57]. MiRNA analysis has defined that ESCs and iPSCs have a distinct miRNA expression patterns, compared to differentiated somatic cells [58]. The miRNA-mediated reprogramming technique has been shown to be more efficient than the standard OSKM over-expression method [59]. Furthermore, cell reprogramming mediated by miRNA may provide a safer approach than traditional DNA reprogramming methods. 

## 8. Stem Cell Function and miRNA Transcription

The mechanisms by which cells control the production and function of miRNAs have been recently described [60]. MiRNAs transcription is regulated like protein coding genes, for example by DNA methylation or through DNA promoter binding factors such as p53 or C-MYC [61]. As previously stated, the factors Oct4, Sox2, and NANOG play a central role in acquiring and maintaining “stemness”: these factors define the future of stem cells through several stages of self and mutual regulation. Recent studies have revealed the transcription of many other actors in the Pluripotency Regulation network, including LIN28, CMYC, Klf4, TCF3, or STAT3 [62]. Some of these nuclear stem cell regulators are able to activate the promoters of different miRNAs in ESCs, including miR-290–295, miR-302–367 and miR-92 clusters [63]. MiRNAs are central players in the process of ESC self-renewal/differentiation. The loss of mir-145 compromises differentiation and induces the expression of Oct4, Sox2 and Klf4 [45]. It also controls the differentiation of ESCs directly by targeting stem cell factors, thus silencing the self-renewal program [45]. MiRNAs involved in maintaining the pluripotency of stem cells are miR-200C and other miRNAs belonging to miR-203 family [46].

## 9. MiRNAs Orchestrating Stem Cell Pluripotency and Differentiation

Studies on mice, revealed that the miR-290–295 cluster and miR-296 were expressed in ESCs and that their levels decrease during stem cell differentiation. This proved that the miR-290–295 cluster had specific roles in maintaining pluripotency [64]. Interestingly, some nuclear stem cell regulators like Oct4 are able to activate promoters of several miRNAs in ESCs, including miR-290–295, miR-302–367, miR-371–373 and miR-92 clusters [63]. While miR-290–295 cluster is highly expressed in mESCs, miR-302–367 and miR-371–373 clusters are mainly regulators in hESCs. Actually, the miR-290–295 cluster represents the mouse homologue of human miR-371–373 [65,66,67,68,69]. The human ESCs express many members of the miR-302 family, as miR-302a, miR-302a*, miR-302b, miR-302b*, miR-302c, miR-302c*, and miR-302d [70]. The overexpression of miR-302 in human cancer cell lines resulted in their conversion into pluripotent cells that express key ESC markers such as Oct3/4, SSEA-3, SSEA-4, Sox2, and NANOG [71]. These miRNAs (miR-302s, miR-369s and miR-200c) are highly expressed in iPSCs and ESCs and are capable of reprogramming human somatic cells to acquire the pluripotent state [72]. Some miRNAs are known to facilitate cellular reprogramming for the generation of iPSCs, while other miRNAs can inhibit this process. This is achieved by reducing directly or indirectly the expression of the pluripotent genes involved in cellular reprogramming. In this regard, miR-34 (miR-34a, b, c) was found to suppress reprogramming of somatic cells by deleting the expression of NANOG, Sox2, and N-Myc [73]. Other differentiation-related miRNAs, as miR-296 and miR-470 were found to target coding regions of NANOG, SOX2 and OCT4 to promote differentiation [74]. The transcriptional effects of miR-21 itself are regulated in ESCs by a transcriptional repressor called the RE1-silencing transcription factor (REST). REST directly interacts with an upstream cis-element of the miR-21-dependent transcription factors, thus promoting differentiation [75]. A recent study demonstrates that miR-21, which is induced following the commitment of mouse ESCs to differentiation, has a potential binding sites in the 3′ UTRs of the mRNAs encoding for NANOG, SOX2, and OCT4, thus modulating the corresponding protein levels [76]. MiRNAs regulation defines ESC fate and behavior (Table 1).

## 10. The Two Big miRNA Families of Pluripotency: miR-200 and miR-302/367

### 10.1. MiRNA-200 Family

The miR-200 family comprises five members (miR-200a, miR-200b, miR-200c, miR-141, and miR-429), located within two clusters on two different chromosomes: chromosomes 1 and 12 in humans [77]. Members of the miR-200 family, directly activated by Oct4 and Sox2 able to bind to their promoter regions, help fibroblasts to overcome the MET (Mesenchymal Epithelial transition) barrier and facilitate iPSC generation. All members of the miR-200 family (miR-200a, miR-200b, miR-200c, miR-141, and miR-429) are significantly more expressed in iPSCs and ESCs than in MEF cells, suggesting that miR-200 family may correlate with pluripotency and that its activation may promote the emergence of iPSCs. Members of the miR-200 family are specific and direct targets for the key pluripotency-associated transcription factors Oct4/Sox2 in iPSC generation. However, very little is known about the role and intrinsic regulation of the miR-200 family during iPSC generation. Oct4 and Sox2 could bind the promoter region of miR-141/200c and miR-200a/b/429, respectively, and activate the transcription of miR-200s. MiR-200 family members have been found to inhibit ESC differentiation directly targeting Cadherin11 and Neuropilin1 [69]. Recently, it has been demonstrated that the MET process is an important early event in iPSC generation [78], and that the activation of EMT is associated with the maintenance of stem-cell properties [79]. Noteworthy, TGF-β induces reversible DNA methylation of miR-200 promoters [80]. Moreover, miR-200b and miR-200c loci can be reversibly de-methylated during prolonged signaling of TGF-β. Changes in the degree of methylation of the miR-200 promoter are closely related to miR-200 expression, and repression. The mechanism through which TGF-β controls the DNA methylation of miR-200 is not clear, but it may involve the presence of active DNA Methyltransferases (DNAMTs). Even invasive mesenchymal breast cancer cell lines show miR-200 methylated promoters, in contrast to epithelial cells where miR-200 promoters were de-methylated. Similar observations have recently been yielded from primary mesenchymal cells and invasive bladder tumors [81,82,83]. Furthermore, Gregorya et al. have identified a central role for an autocrine TGF-β/ZEB/miR-200 signaling network in controlling the transition between epithelial and mesenchymal states. Prolonged activation of this pathway leads to epigenetic changes in miR-200 and may contribute to invasive breast cancer progression [84]. Considering these findings, a remarkable connection between EMT and breast cancer stem cells was recently demonstrated, where TGF-β treatment was shown to initiate EMT-associated embryonic differentiation programs of epithelial–mesenchymal, with EMT and MET representing a basic mechanism for epithelial cell plasticity [79]. MET has been shown to be an important early event in somatic cell reprogramming [76,77,78,79,80,81,82,83,84,85]. The miR-200 family and ZEB1 have been shown to be key regulators of these processes [85,86]. A link among the TGF-β–related factors, the bone morphogenetic proteins, and the miR-200 family have recently been described in somatic cell reprogramming [87]. It is well known that the miR-200 family suppresses EMT. It has also been reported that the knockdown of miR-200c inhibits NANOG and up regulates GATA4, thereby inhibiting self-renewal and promoting hESC differentiation [88]. Transfection with a group of miRNAs including miR-200c, miR-302s, and miR-376s family successfully coaxed somatic cells into an iPSC fate [89]. As stated above, TGF-β plays an important role in repressing the miR-200 [90].

### 10.2. The miR-302/367 Family

The miR-302/367 big family, comprises miR-367, miR-302d, miR-302a, miR-302c, miR-302b, and miR-371/373. The miR-302 cluster is the most abundant miRNA family in hESCs, and can promote somatic cell reprogramming, while miR-371–373, are less expressed [91]. The structure of the gene coding for the human miR-302/367 cluster has been well characterized [92]. MiR-371, miR-372, and miR-373 are gene clusters located in the region of the human chromosome 19q13.4. They are the human equivalent of miR-290/295 in the mouse [93]. MiR-302/367 family is ubiquitously distributed in vertebrates as a cluster spanning the La-related protein 7 (LARP7) intron, in the 4q25 region of human chromosome 4 [92]. The cluster was initially identified to be specifically expressed in undifferentiated hESCs, and their malignant counterpart human embryonic carcinoma cells (hECCs), and was found to play a role in pluripotency of stem cells and in cancer formation [92]. This cluster is highly expressed in, and therefore constitutes a signature miRNA for stem cells [93,94]. It is also highly expressed in iPSCs while progressively declining during differentiation [93]. The miR-290–295/miR-371–373 family members directly control the G1-S cell cycle transition and inhibit apoptosis. This family also plays a significant role in regulating cell proliferation, differentiation and reprogramming [95,96]. It was demonstrated that Oct3/4, NANOG, Rex1, and Sox2 act as transcriptional activators of the miR-302/367 cluster [97,98]. This cluster is specifically expressed in embryonic stem cells, iPSCs or tumor cells, to coordinate proliferation, differentiation, pluripotency, maintenance, and reprogramming [99,100]. MiR-302 members repressed lysine-specific histone demethylase 1 and 2 (AOF1 and AOF2) and methyl-CpG binding proteins (MECP1 and MECP2), leading to destabilization of DNA methyltransferase 1, thus causing a genome-wide demethylation promoting reprogramming and iPSC development [101]. Overexpression of the miR-302-367 cluster significantly increased the conversion of reprogrammed iPSCs by repressing MBD2 (methyl-CpG binding domain) expression, thereby inducing NANOG expression [102].

Another study found that the miR-302/TGF-β/Nodal/Smad-2/3 pathway was also involved in EMT. In EMT, epithelial cells form monolayers and display elongated morphology and enhanced motility [103]. MiR-302 members directly repressed the expression of the transforming growth factor beta receptor 2 (TGFBR2), and the member C (RHOC) genes of the Ras family, thus allowing the EMT process [92]. In addition, miR-302 members could negatively regulate the level of the TGF-β related protein Lefty1 and Lefty2, thus becoming an upstream modulator of the TGF-β/Nodal signaling pathway, finely tuning pluripotency and differentiation [104]. The main core promoter for this process has been shown to include the embryonic stem cell factors NANOG, OCT3/4, SOX2, and REX1 [105]. This is an additional cue to consider the miR-302/367 cluster an important stem cell signature for stem cells [92]. This cluster is also highly expressed in iPSCs where its expression decreases during differentiation [105].

## 11. Epithelial–Mesenchymal Transition (EMT) in Stem Cells

TGF-β plays an important role during cell reprogramming in iPSC generation and pluripotency. Fibroblasts undergoing MET adopt the epithelial state of pluripotent cells and conversely human pluripotent stem cells resemble cells obtained from the epiblast [106]. The occurrence of EMT in human pluripotent stem cells and embryoid bodies during cell commitment reflects the EMT observed during gastrulation in human development [107]. By contrast, suppression of EMT by inhibiting TGF-β signaling improves reprogramming efficiency [108]. The TGF-β signaling pathway promotes EMT, and miR-302 promotes reprogramming partly decreasing the expression of TGF-β receptor 2, and the related SMAD2 and/or SMAD3 phosphorylation and activation [109]. Furthermore, as stated above, TGF-β plays an important role in repressing the miR-200. Members of the miR-200 family are specific and direct targets for the key pluripotency-associated transcription factors Oct4/Sox2 in iPSC generation. These miRNAs act as regulators of EMT in many cell types. For example, snail promotes EMT through the repression of miRNA 200 family members and induces mesoderm differentiation during the epiblast stem cell stage, suggesting that miR-200 family members might suppress EMT and the differentiation of ESCs at the epiblast stem cell stage [67]. These two major miRNAs families are the basis of the pluripotency induction by interacting with the TGF-β, thus demonstrating that TGF-β plays a crucial role in suppressing pluripotency by acting on the EMT [106]. Among the other TGF-β may be triggered by ROS (reactive oxygen species) [50].

## 12. Hypoxia Exerts Opposite Effect on miR-200 and miR-302 Family

Hypoxia is broadly recognized as a mechanism that drives angiogenesis. Several possible mechanisms have been proposed for down-regulation of miRNAs in a low oxygen environment [110]. MiR-200b down regulation is crucial in inducing angiogenesis via Ets-1 direct targeting. These findings consolidate the notion that under hypoxic conditions miR-200b down-regulation is required to relieve Ets-1 repression resulting in successful angiogenic outcomes. Furthermore, hypoxia-induced angiogenesis, as well as Ets-1 up-regulation was rescued by delivery of the miR-200b mimic [110]. On the other hand, miR-302 expression was induced by hypoxia. Recent findings by other Authors highlighted a central role of miR-302 overexpression during hypoxia/re oxygenation-mediated cardiomyocyte apoptosis. In particular, this proapoptotic effect of miR-302 was mediated by inhibiting the expression of the antiapoptotic molecule myeloid leukemia cell-differentiation protein-1 (Mcl-1) [111].

## 13. Physical Stimuli: Ad Hoc Conveyance of Electromagnetic Fields to Drive Stem Cell Fate

**Figure 1:**
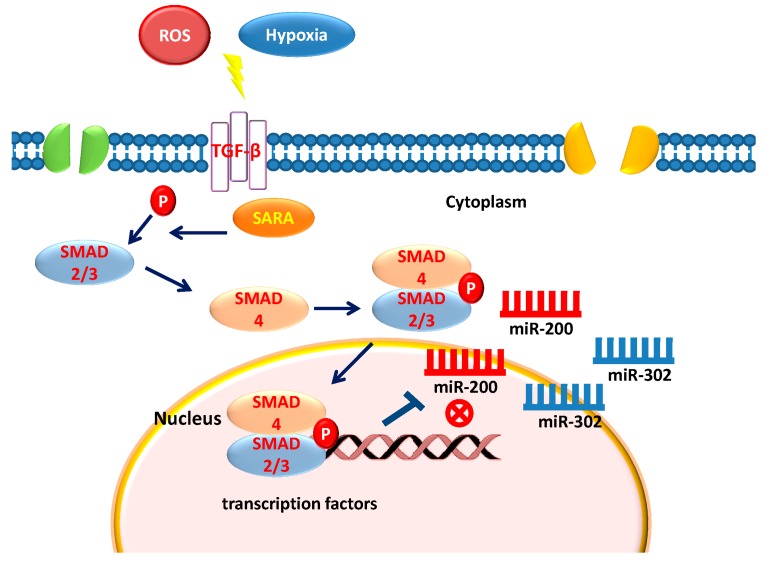
TGF-B activation and SMAD-mediated miRNA downregulation.

Previous studies revealed a role of reactive oxygen species (ROS) in transducing mechanically or electrically-induced cardiovascular differentiation in mouse ESCs [112]. Moreover, fibroblast direct reprogramming toward cardiogenic, neurogenic, and myogenic lineages occurred through an upregulation of the NADPH oxidase isoforms Nox2 and Nox4 [113]. In the same paper, Maioli et al. described an interesting biphasic effect on the expression of certain stemness related genes, elicited by the Radio Electric Asymmetric Conveyer (REAC), an innovative device conveying radio electric fields of 2.4 GHz. In particular, REAC-stimulation was able to induce an increase in the expression of Oct4, Sox2, cMyc, NANOG, and Klf4 within 6–20 h, while downregulating the same genes following prolonged times of exposure [113,114]. This study demonstrates the feasibility of using a physical stimulus to afford the expression of stemness in human adult somatic cells up to the attainment of three major target lineages (cardiogenic, neurogenic, and skeletal myogenic) (Figure 1). So far, the acquirement of a pluripotent state in somatic cells, including fibroblast reprogramming to iPSCs or direct reprogramming to cardiac or neuronal lineages without an iPSC intermediate, has only been achieved by targeted genetic engineering through viral vector-mediated technologies or cumbersome and expensive protein transduction methods or by combinatorial approaches encompassing both viral-mediated gene delivery and chemical stimulation or gene-delivery and physical stimulation of the target cells [114,115,116]. Electromagnetic fields (EL-EMFs) are also able to affect biological processes, including stem cell development proliferation and differentiation [117].

## 14. Electromagnetic Fields

Baek et al. showed that EMF exposure induces epigenetic changes that promote efficient somatic cell reprogramming to pluripotency [118]. There is a general agreement on the effects of EMF on biological systems. It was indicated that low-frequency electromagnetic fields affect cell migration, cellular differentiation, apoptosis, and stress response [119,120]. EMF can also affect many stages of embryonic development, which are of interest for morphology and migration of embryonic cells [120,121]. Moreover, it was reported that EMFs of selected frequencies promote osteogenic and neurogenic differentiation, a finding that has been clinically applied in repairing bone fractures and promoting wound healing [122,123,124]. These studies indicate that EMFs can be involved in managing the conversion of cell destiny. Stem cells respond to EMFs differently, depending on their state of differentiation. It is possible that EMFs modulate the activity of transcription factors and the level of cell cycle regulatory genes [125,126,127]. It is believed that one of the possible mechanisms of EMF biological activity involves the generation of ROS within the cell. Excessive concentration of ROS, such as superoxide anions (O_2−_) and hydrogen peroxide (H_2_O_2_), is considered to be cell destructive and results in inhibition of gene expression. In contrast, small amounts of ROS function as intracellular second messengers and activate signaling cascades involved in growth and differentiation of many cell types. The high level of ROS modifies signaling pathways by phosphorylation mechanisms [128]. However, the energy of a weak EMF is not sufficient to directly break a chemical bond in DNA [128]. Therefore, it can be concluded that genotoxic effects are mediated by indirect mechanisms as microthermal processes, generation of ROS, or disturbance of DNA repair processes [128]. Although ROS were originally thought to be merely a harmful product of metabolism, accumulating evidence demonstrates a role of ROS in cell fate signaling [128]. H_2_O_2_ is thought to be the main ROS species involved in intracellular signaling, and in specific contexts can act directly as a second messenger, integrating environmental cues and passing them to downstream signal transduction cascades [128,129]. This is due mostly to the longer half-life of H_2_O_2_ and its ability to diffuse easier through membranes than other types of ROS [129,130]. Slight variations in ROS content may have deep effects on stem cell fate [130,131]. iPSCs and ESCs exhibit similar ROS levels and mitochondrial mass, but significantly less than in human dermal fibroblasts from which iPSCs were generated. As stated before, the loss of mitochondria appears to be a key step in the reprogramming process, and is linked to a switch from an oxidative metabolism to a glycolytic-dependent state [132,133]. It has been recently shown that maintaining low (physiological) O_2_ (2–5% O_2_) in cultures inhibits spontaneous differentiation and supports pluripotency [132,133,134]. These studies provided evidence that molecular processes that mediate pluripotency and suppress differentiation are supported under 4%-O_2_ conditions and that the cells retain a “memory” of their prior environment. Hypoxia induces the expression of Sox2 and Oct4 genes that are related to stem cell function [135,136]. In particular, Sox2, together with Sox4, was recently shown to play a pivotal role in the maintenance of stemness in ESCs. Hypoxia has been demonstrated to induce EMT, which prompts invasion and metastasis from cancer cells [135]. During EMT, epithelial cells undergo several biochemical alterations that allow the acquisition of the mesenchymal phenotype enabling cancer cells to evade their “homeland” and to colonize remote locations [135,136]. EMT-inducers, including TGF-β and hypoxia, trigger changes in gene expression by complex signaling pathways [136]. The loss of polarity and gain of motile characteristics of mesenchymal cells during embryonic development have suggested analogies with metastatic cancer cells during malignant progression [135]. Notably, recent data on several cancer types have demonstrated that EMT is involved in generating cells with properties of stem cells [136]. This implies that hypoxia-induced EMT may affect cancer stem cells (CSCs) or induce stem-like cells from more differentiated progenitors determining an increase of CSC population responsible for early systemic cancer dissemination and metastasis formation. Many studies have demonstrated a functional connection between low oxygen level, ROS production, and EMT [134,137]. Indeed, in contrast to cancer cells in which ROS levels are increased, CSCs generally maintain low ROS, exhibiting redox patterns that are similar to the corresponding normal stem cells [137]. Diehn et al. reported that ROS levels are lower in human and murine breast CSCs than in non-stem breast cancer cells [135].

## 15. ROS and miRNA

The miR-200 family has been extensively studied in the EMT of cancer cells [30]. In EMT, miR-200 family down-modulation enhances cancer aggressiveness and metastases, whereas reintroduction of miR-200 family in some tumors inhibits their growth. ROS induces miR-200c and other miR-200 family members; the following down-modulation of ZEB1 has a key role in ROS-induced apoptosis and senescence [138]. Another study demonstrated that H_2_O_2_ and other oxidant agents increase the expression of miR-200c and induce growth arrest, apoptosis, and senescence in HUVEC cells by inhibition of ZEB1 expression [138]. Overall, these findings indicate a potential role of miR-200 family in the regulation of ROS homeostasis [138]. The effect of miR-302s on cell survival under oxidative stress has been shown to determine the effects of miR-302d on ROS, with a protective role against oxidative stress [139]. Accordingly, miR-302d transfection significantly decreased the generation of ROS, indicating that the protective action of miR-302 on oxidant-induced cell death may be related to the inhibition of ROS generation [140].

As mentioned above, the interaction of miR-200 and miR-302 families with ROS is different. Mir-200 family members increase after ROS stimulation, while miR-302 family act by lowering the level of ROS. There are therefore opposite mechanisms that maintain cellular homeostasis and that can be used to modulate pluripotency and differentiation in stem cells [138,139,140,141]. The mechanisms that induce pluripotency and differentiation are complex, and not fully clarified. The interest of the scientific world has increased since Yamanaka created iPSCs, paving a novel way to regenerative medicine. To date, the limits of using iPSCs for regenerative purposes are caused by the use of viruses to transport OCT4, SOX2, c-Myc, NANOG, or other pluripotency factors within the target. This can cause teratoma and possibly cancer.

On the whole, our interest in writing this review was to understand how miRNAs work in stem cells, cancer stem cells, or iPSCs. We also attempted in drawing the readers’ attention on transcription factors, and physical stimuli that can be used to maintain pluripotency, or to induce differentiation.

## 16. Conclusions

**Figure 2:**
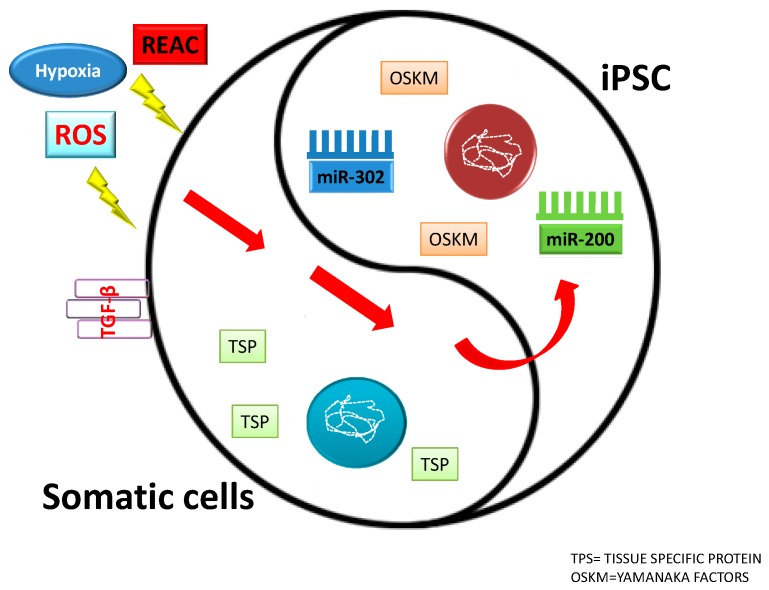
Influence of different factors on somatic cells and induced pluripotent stem cells (IPSCs) fate.

**Figure 3:**
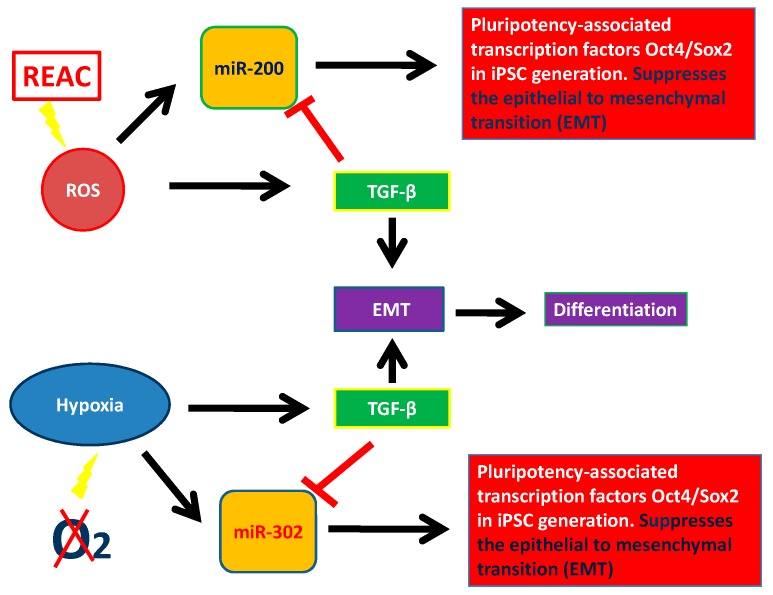
Interaction between miR-200, miR-302, physical stimuli, TGF-β and pluripotency.

In this review, we described epigenetic factors modulating pluripotency and stem cell behavior, particularly the role of miRNAs. We considered two large miRNA families involved in plutipotency: miR-200 family and miR-302 family. We analyzed some features of stem cells which encompass miRNA recruitment, biophysical stimuli, such as electromagnetic fields and REAC conveyed radioelectric fields, and chemistry, like ROS and hypoxia. The REAC action bypassed a persistent reprogramming toward an induced pluripotent stem cell-like state and involved the transcriptional induction of the NADPH oxidase subunit Nox4 [112,113,114,115]. This finding demonstrates the feasibility of using a physical stimulus to afford the expression of pluripotentiality in human adult somatic cells up to the attainment of major complex lineages. It is believed that one possible mechanism involves the generation of ROS within the cell. Excessive concentration of ROS is considered to be cell destructive and results in inhibition of gene expression. In contrast, small amounts of ROS function as intracellular second messengers and activate signaling cascades involved in growth and differentiation of many cell types. This complicated regulatory mechanism is called “hormesis”. Hormesis can be considered an adaptive function characterized by a dose-dependent biphasic response, which occurs as a result of exposure to a very wide range of stimuli [142,143,144]. This intricate mechanism of activation and inhibition of miRNAs and their targets leads to a fine stem cell homeostasis. MiR-200 and miR-302 are pluripotency activators which trigger pluripotency genes (SOX2, OCT4, and NANOG). These two miRNAs families can be modulated by physical and chemical factors. As showed in Figure 2, ROS induction leads to TGF-β activation which acts blocking miR-200 and miR-302. Figure 2 shows the complex interplay occurring between ROS, miR-200, and TGF-β and between hypoxia, miR302, and TGF-β. This factor, is directly modulated by both of the above mentioned miRNAs, and acts itself by modulating cell differentiation and reprogramming, in turn downregulating both miRNA 200 and miRNA 302. These two major miRNA families are the basis of pluripotency induction by interacting with TGF-β, thus demonstrating that TGF-β, with or without the involvement of ROS, plays a crucial role in suppressing pluripotency, thus influencing stem cell behavior (Figure 3) [112]. Unravelling the hormetic factors, as for example ROS, and their role in influencing stem cells pluripotency and differentiation capabilities could pave the way to novel therapeutic approaches in regenerative medicine.

## Figures and Tables

**Table 1:** MiRNA regulation on stemness.

miRNA	Influence on Pluripontecy	Influence on Differentiation	Inhibition of EMT /MET
miR-200 family	yes	no	yes
miR-302 family	yes	no	yes
